# The use of internet for health information by hospitalised patients in Switzerland and Qatar: a comparative cross-sectional study

**DOI:** 10.1080/20523211.2025.2594827

**Published:** 2025-12-05

**Authors:** Ameena Jesaimani, Jassim Fakhro, Youssef Daali

**Affiliations:** a Department of Pharmacy, Hamad Medical Corporation, Doha, Qatar; b Surgery Department, Metabolic, bariatric, and robotic surgery, Hamad Medical Corporation, Doha, Qatar; c Department of Clinical Pharmacology and Toxicology, Geneva University, Geneva, Swtizerland

**Keywords:** Internet, health information, hospitalized patients, Switzerland, Qatar, questionnaire survey

## Abstract

**Background:**

The internet has become a critical resource for accessing health information worldwide. Online health information seeking (OHIS) is increasingly common among hospitalised patients, particularly those with chronic conditions. While prior studies have explored OHIS in individual countries, limited evidence exists comparing behaviours across different sociocultural and healthcare contexts. This study compared patterns of internet use for health information among hospitalised patients in Switzerland and Qatar.

**Methods:**

A comparative cross-sectional study was conducted between January and June 2016 in two tertiary hospitals in Switzerland, and Qatar. Eligible patients (18–80 years) admitted to internal medicine, visceral surgery, or orthopaedics wards completed a 33-item structured questionnaire, available in French and Arabic, covering sociodemographic characteristics, internet access, health information-seeking behaviour, and use during hospitalisation. Both descriptive and inferential statistics were applied to identify predictors of OHIS.

**Results:**

A total of 820 patients participated (617 Swiss, 203 Qatari). Swiss patients were older (mean age 57 ± 15) than Qataris (44 ± 16, *p* ≤ 0.001). Qatari patients were more likely to search for health information online compared with Swiss patients (85% vs 65%, *p* ≤ 0.001). They searched more frequently for information on diseases (74% vs 56%), treatments (59% vs 41%), healthcare professionals (40% vs 20%), and hospitals (41% vs 18%). Online health information had greater reported impact among Qataris, prompting them to ask further questions to doctors (75% vs 50%, *p* ≤ 0.001) and influencing decisions to consult physicians (33% vs 22%, *p* ≤ 0.05). Both cohorts expressed interest in reliable, tailored online resources, with Qataris showing stronger preference for interactive and video-based platforms.

**Conclusion:**

This study highlights significant cross-country differences in OHIS behaviour among hospitalised patients, shaped by sociodemographic, cultural, and healthcare system contexts. Findings underscore the need for culturally relevant, trustworthy, and patient-centred digital health resources to enhance patient empowerment, improve clinician–patient communication, and reduce risks of misinformation.

## Background

Over the past decade, the internet has experienced unprecedented growth, both in technological sophistication and accessibility (Vakamullu, 2025). The global landscape of internet usage has evolved dramatically from the 3.1 billion users in 2015 to 5.6 billion in 2025 (Kirksekiz et al., 2025; Union, 2012). Internet use for health information is widespread, with approximately 58.5% of U.S. adults and around 55% of Europeans seeking medical information online (Geller et al., 2025; Pierrakos et al., 2023). In contrast, internet usage for health information in the Middle East shows considerable variation, ranging from as low as 15% in Saudi Arabia, to 71% among the Qatari population and less than 50% among participants in other middle eastern regions including Jordan and Kuwait (Alhuwail & Abdulsalam, 2019; Alhuthail et al., 2025; Al-Ruzzieh et al., 2024).

Online health information seeking (OHIS) is common among hospitalised patients, notably among those with chronic illnesses, and their treatment demands understanding specific information such as diseases causes, test results, treatment options, and management strategies (Zhang et al., 2022). Since online sources are easily accessible and convenient compared to formal healthcare services, patients often engage in online health information seeking. While accessing health information using internet has become increasingly convenient and can enhances individual’s knowledge and confidence, however, the information obtained requires careful evaluation and critical appraisal before it can be reliably utilised (Dahiyat et al., 2023a; Di Novi et al., 2024; Showande & Laniyan, 2022).

Several studies have examined factors influencing OHIS behaviour, a recent systematic review and meta-analysis used multiple theoretical models reported perceived quality and trust on online information as key predictors of OHIS (Wang et al., 2021). A large-scale study from China involving more than 10,000 participants reported that although OHIS does not directly influence the individual’s health behaviour, but instead, the perceived information quality acts as bridge between online information seeking behaviour and subsequent health actions (Liu et al., 2024). Limited access to and utilisation of internet is also linked to suboptimal health management and lower self-rated health status, however, greater access to online resources has been associated with improved health outcomes (Liu et al., 2024).

Furthermore, Internet access provides opportunities for patients to access additional information, clarify their understanding, and prepare questions for their treating physicians (Clarke et al., 2016; Dahiyat et al., 2023; Lu, 2023). Anecdotal evidence suggests that online resources used during hospitalisation can influence patient–clinician communication, satisfaction, and decision-making (Laugesen et al., 2015; Luo et al., 2022). Recent studies on OHIS behaviour have primarily focused on characteristics such as population groups, age categories, varying health status. To further illustrate the diverse context of OHIS behaviour, several studies have also reported findings on patients with specific diseases such as cancer, hypertension, cardiac issues, type 2 diabetes mellitus, and rare disease (Freeman et al., 2018; Jamal et al., 2015; Stanarević Katavić, 2019; Zhao et al., 2022). Nevertheless, limited information is available regarding how OHIS behaviour varies across different healthcare landscapes, socio-economic and socio-cultural contexts, notably when comparing populations in two different setting such as Qatar and Switzerland.

This study provides one of the first cross-country comparisons of internet usage for seeking online health information seeking among hospitalised patients, using cohorts from Switzerland and Qatar. By examining two settings with distinct sociocultural, linguistic, and healthcare characteristics, the findings highlight how digital infrastructure, language accessibility, and cultural expectations shape patients’ engagement with online health information. Importantly, our findings are anticipated to inform whether patients’ access to reliable and comprehensible online resources during hospitalisation may influence patient empowerment, improve communication with healthcare providers, and support more informed decision-making. The primary objectives of this were to assess and compare patterns of Internet use for health information among hospitalised patients in these two distinct sociocultural contexts.

## Methods

### Study design and setting

This was a comparative cross-sectional study conducted between January and June 2016 in two tertiary healthcare institutions: Geneva University Hospitals (HUG) in Switzerland and Hamad General Hospital (HGH) in Qatar. HUG is one of the largest academic medical centres in Switzerland, serving a diverse population, while HGH is a leading referral hospital in Qatar, providing care to a multi-ethnic patient population in a rapidly developing healthcare system.

### Study population

Hospitalised patients of age 18–80 years, admitted to internal medicine, visceral surgery, or orthopaedics wards were included. Inclusion criteria required patients to be physically and cognitively capable of providing informed consent and completing the questionnaire. Only patients fluent in French (Switzerland) or Arabic (Qatar) were included. Paediatric patients, and patients admitted to intensive care, those with severe medical or psychiatric conditions, or those unable to communicate effectively were excluded from participation.

### Sample size

The sample size was calculated based on an estimated prevalence of Internet use for health information of 30%, with a 95% confidence level and a margin of error of 5%. After adjusting for an anticipated non-response rate of 20%, the target sample was set at 400 patients per site, for a total of 800 participants.

### Data collection tool

A structured, self-administered questionnaire was developed in both French and Arabic to capture relevant data for the study. It consisted of 33 items covering five key domains. The first domain focused on general Internet use, including frequency of use, devices accessed, and purposes for which the Internet was utilised. The second domain examined health information-seeking behaviour, exploring prior use of the Internet to search for health-related content such as diseases, treatments, and medications, along with perceptions of the reliability and usefulness of the information obtained. The third domain assessed Internet use during hospitalisation, addressing access to devices and hospital Wi-Fi, the types of health information sought while admitted, and any influence of this information on interactions with healthcare providers or patient decision-making. The fourth domain collected sociodemographic data, including age, gender, marital status, education level, employment status, and presence of chronic health conditions. Finally, the fifth domain investigated health and medication details, documenting current diagnoses, regular medications, and whether patients sought online information related to these conditions.

The questionnaire was adapted from previously validated instruments, including the Pew Internet Project survey and tools from the California HealthCare Foundation (Pennbridge et al., 1999). A pilot test was performed with four patients in Geneva to assess clarity and relevance. The Arabic version underwent back-translation to ensure linguistic and conceptual equivalence. Average completion time was approximately 30–45 min.

### Data collection procedure

Trained research assistants (two) approached eligible patients during their hospital stay, explained the study purpose, and obtained informed consent. Both research assistants were well trained and followed a standardised protocol and uniform patient approach, with periodic supervision to minimise variability and potential bias. Participants could complete the questionnaire independently or request assistance if needed. Each respondent was assigned an anonymised code to maintain confidentiality.

All data were securely stored on password-protected computers with access restricted to the principal investigators and designated research assistants. To ensure confidentiality, patient identifiers were removed prior to analysis. The anonymised dataset remains securely archived and is available upon reasonable request.

### Ethical considerations

Ethical approvals were obtained from the institutional review boards of Geneva University Hospitals and Hamad Medical Corporation. Participation was voluntary, and no incentives were offered. All responses were kept confidential, and no identifiable patient information was collected.

### Data analysis

Data were entered and analysed using IBM SPSS Statistics version 23 (IBM Corp., Armonk, NY, USA). Descriptive statistics (means, standard deviations, frequencies, and percentages) were used to summarise patient characteristics and patterns of Internet use. Differences between groups were assessed using Chi-square tests for categorical variables and independent samples t-tests for continuous variables. Statistical significance was set at *p* < 0.05.

## Results

A total of 820 hospitalised patients (617 Swiss and 203 Qatari) were included in the study. The sociodemographic and health status characteristics of these patients are summarised in Table 1.

**Table 1.** Sociodemographic and health status characteristics of hospitalized Swiss and Qatari patients.

Items	Categories	Swiss	Qatari	P-value
Gender	Female	273 (44%)	100 (49%)	<0.001
	Male	344 (56%)	103 (51%)	
Age	Mean (SD)	57 (15)	44 (16)	<0.001
Marital status	Single	151 (24%)	46 (22%)	<0.001
	Married	269 (44%)	129 (64%)	
	Divorced	144 (23%)	8 (4%)	
	Widowed	53 (9%)	20 (10%)	
Educational status	High school	484 (78%)	101 (71%)	<0.001
	University	133	58 (29%)	
**Patient Health Status**				
Chronic diseases	Hypertension	142 (23%)	63 (31%)	≤.001
	Heart disease	83 (14%)	19 (9%)	≤.001
	Pulmonary problems	78 (13%)	19 (9%)	≤.001
	Diabetes	83 (14%)	58 (29%)	≤.001
	Cancer	90 (15%)	2 (1%)	≤.001
	Pain	167 (27%)	22 (11%)	≤.001
	Other chronic problems	120 (19%)	24 (12%)	≤.001
	No chronic problems	194 (31%)	87 (43%)	≤.001
Chronic medicine intake	Yes	371 (60%)	104 (51%)	>0.05
	No	246 (40%)	99 (49%)	
Length of hospital stay	Mean (SD)	10 (14)	10 (12)	
**Internet Use**				
Most frequently used electronic devices	Desktop	237 (38%)	67 (33%)	>0.5
	Laptop	297 (48%)	125 (62%)	
	Smartphone	203 (33%)	105 (52%)	
	Electronic tablet	98 (16%)	76 (37%)	
Frequency of internet use	At least once a day	299 (49%)	97 (48%)	>0.5
	Less frequent	89 (15%)	44(22%)	
	Never	212 (35%)	62(31%)	

### Socio-demographic characteristics

The demographic profiles of the two cohorts showed significant differences (Table 1). In Switzerland, a higher proportion of male participants was observed (56%) in comparison to females (44%). In contrast, gender distribution in Qatar was nearly equal (male: 51%, female: 49%). The mean age of Swiss patients was significantly higher at 57 ± 15 (17–82) than Qatari patients, who had a mean age of 44 ± 16 (18–80), (*p* ≤ 0.001).

Marital status differed notably between groups. While the majority of participants in both countries were married, this proportion was significantly higher among Qataris (64%) compared to Swiss patients (44%) (*p* ≤ 0.001). A higher percentage of Swiss patients were divorced (23% vs 4%) or widowed (9% vs 10%). Education levels also revealed disparities. Qatari patients were more likely to have completed only obligatory schooling (43% vs 28%), while Swiss participants exhibited a broader educational distribution, with greater proportions having completed high school (26%) and university degrees (22%) (*p* ≤ 0.001).

### Health status and chronic conditions

Differences in health profiles were apparent between cohorts (Table 1). Nearly half (43%) of Qatari patients reported no chronic health conditions compared to 31% of Swiss patients (*p* ≤ 0.001). Among those with chronic illnesses, Swiss patients were more likely to suffer from chronic pain (27% vs 11%), heart disease (14% vs 9%), pulmonary problems (13% vs 9%), and cancer (15% vs 1%). Conversely, hypertension (31%) and diabetes (29%) were more prevalent among Qatari patients (*p* ≤ 0.001 for all comparisons).

The use of chronic medication was also significantly higher among Swiss patients (60%) compared to Qataris (51%) (*p* ≤ 0.05). The average hospital stay at the time of the interview was comparable between groups (mean 10 days in both), and over half of participants in both cohorts were admitted via the emergency department.

### Health information-seeking behaviours

Health information seeking emerged as a central motivation for internet use, particularly among Qatari patients (Table 2). Significantly more Qataris searched for health information online (85% vs 65%, *p* ≤ 0.001). Specific topics sought by Qatari patients included diseases or medical problems (74% vs 56%), treatments or interventions (59% vs 41%), information about doctors or other healthcare professionals (40% vs 20%), and hospitals or medical facilities (41% vs 18%).

**Table 2.** Health information seeking behavior of hospitalised patients.

Items	Categories	Swiss	Qatari	P-value
Reasons for internet use	Professional	219(58%)	85(57%)	ns
	Entertainment	258(68%)	109(74%)	ns
	General information	317(83%)	119(80%)	ns
	Health information	247(65%)	125(85%)	≤.001
	Video	171(45%)	97(66%)	≤.001
	Online shopping	163(43%)	55(37%)	ns
Types of searches	A specific disease	211(56%)	108(74%)	≤.001
	Treatment/intervention	152(41%)	86(59%)	≤.001
	Doctors or other health professionals	74(20%)	59(40%)	≤.001
	Hospital or other medical facilities	69(18%)	60(41%)	≤.001
	Health insurance	80(21%)	17(12%)	≤.001
	Environmental risk	67(18%)	35(24%)	≤.01
	Food risk	98(26%)	64(44%)	ns
	Medication risk	98(26%)	63(43%)	≤.001
	Chronic pain	46(12%)	26(18%)	≤.001
	Medical examination	29(8%)	39(27%)	ns
	Prescription or over the counter	81(22%)	28(19%)	≤.001
	Complementary and alternative therapy	62 (17%)	72 (49%)	ns
	Diet/ Vitamins	87 (23%)	73(50%)	≤.001
	Never searched for health information	136 (36%)	22(15%)	≤.001
Common search engines/websites used for health information	Web browsers (such as a Google or Internet Explorer etc.)	219(90%)	108(88%)	ns
	Specific health-based websites (such as WebMD, MedlinePlus etc.)	25(6%)	13(9%)	≤.001
	Don’t know	7(3%)	2(2%)	ns

### Common reasons for internet search and searched topics

A focused analysis of internet use during hospitalisation revealed additional insights into patient behaviours and preferences (Table 2). Awareness of hospital Wi-Fi availability was significantly higher among Qatari patients (67%) compared to Swiss patients (48%, *p* ≤ 0.001). When asked about their reasons for internet use during hospitalisation, the most frequently reported purpose was searching for general information 83% among swiss citizens vs 80% Qataris followed by entertainment, cited by 68% of Swiss and 74% of Qatari patients. Other reasons included seeking general information (Swiss: 60%, Qataris: 47%), and health-related information (Swiss: 42%, Qataris: 49%). When exploring health-related searches during hospitalisation, only 42% of Swiss patients and 50% of Qatari patients reported seeking such information. Among those who searched for health information, the most common topics included diseases or medical problems related to current hospitalisation (Swiss: 30%, Qataris: 36%, *p* = ns), treatments or procedures currently being received (Swiss: 14%, Qataris: 20%, *p* = ns), alternative or complementary treatments (Swiss: 4%, Qataris: 11%, *p* = ns), and drugs administered during hospitalisation (Swiss: 14%, Qataris: 6%, *p* = ns).

### Impact of online health information

The perceived impact of online health information on patient behaviour and decision-making was pronounced among Qatari participants (Table 3). A significantly greater proportion of Qataris reported that online health information prompted them to ask doctors further questions (75% vs 50%, *p* ≤ 0.001), influenced their decision to consult a physician (33% vs 22%, *p* ≤ 0.05), and led them to inquire with other healthcare professionals (27% vs 16%, *p* ≤ 0.01).

**Table 3.** Health impact of online health information on Swiss versus Qatari patients.

Items	Categories	Swiss	Qatari	P-value
Perceived Impact of Online Health Information on Health-Related Decisions	Influenced a decision about the treatment of health problems concerning you	63(26%)	39(32%)	ns
	Influenced a decision about the treatment of health problems concerning your relatives or friends	20(8%)	41(33%)	<0.001
	Lead to change in management of chronic illness	42(17%)	27(22%)	ns
	To be healthier (e.g. diets, physical exercise)	81(34%)	63(51%)	≤.001
	Led to better pain management	27(11%)	17(14%)	ns
	Influenced your decision to consult a doctor	53(22%)	41(33%)	<0.5
	Encouraged you to ask a doctor further questions	120(50%)	93(75%)	≤.001
	Encouraged you to ask another professional healthcare worker	38(16%)	34(27%)	≤.01
	Caused change in medical treatment	19(8%)	32(26%)	≤.001
	No influence	56(23)	9(7%)	≤.001
Perceived Health Outcomes	Positive	94(39%)	88(71%)	<0.001
	Negative	7(3%)	14(11%)	
	No influence	123(51%)	11(9%)	
	Don’t know	16(7%)	11(9%)	

Both groups expressed satisfaction with the information found online, although this was slightly higher among Swiss patients (72% vs 69%). However, negative emotional responses such as feeling overwhelmed (52% vs 34%, *p* ≤ 0.001) was more frequently reported by Qatari patients.

### Patient preferences for future online resources

When asked about desired features of future online health platforms, Qatari patients expressed a stronger preference for interactive and secure websites that allow queries regarding medication (83% vs 59%, *p* ≤ 0.001) and side effects (76% vs 56%, *p* ≤ 0.01). Furthermore, Qataris indicated greater interest in receiving specific information on their prescribed medications (87% vs 36%, *p* ≤ 0.001) and videos related to their health conditions (72% vs 47%, *p* ≤ 0.001).

## Discussion

### Statement of key findings

A total of 820 hospitalised patients participated in the study, comprising 617 Swiss and 203 Qatari individuals. The two cohorts differed significantly in their sociodemographic and health profiles. Swiss patients were notably older (mean age 57 ± 15) than their Qatari counterparts (mean age 44 ± 16). Although the initial target was 400 patients per site, the final sample differed from the actual calculation. In Switzerland, recruitment proceeded smoothly and exceeded the target, whereas in Qatar, logistical constraints, language barriers, and lower participation willingness contributed to reduced enrolment. Marital status and education also varied, with a greater percentage of Qatari patients being married and having completed only schooling, whereas Swiss patients showed higher rates of divorce, widowhood, and advanced education. Health status differences were apparent; Swiss patients reported more chronic conditions, including pain, cancer, pulmonary and heart diseases, whereas Qatari patients had higher rates of hypertension and diabetes. Despite these contrasts, the average hospital stays, and emergency admission rates were similar across both groups. Health information-seeking behaviours revealed that Qatari patients were significantly more active in searching for online health information, particularly about diseases, treatments, healthcare providers, and hospitals. Online resources had a stronger impact on Qatari patients, prompting more engagement with healthcare professionals, although they also experienced more negative emotional responses such as feeling overwhelmed. Finally, Qatari patients expressed a clear preference for interactive, secure digital platforms offering tailored information on medications and conditions, highlighting the growing demand for culturally and contextually relevant health information tools in the region.

Conducting the study in both Switzerland and Qatar, two countries with contrasting healthcare systems, patient populations, and cultural contexts, offers a unique opportunity to examine whether patterns of internet use for health information are context-specific or consistent across diverse settings. This cross-cultural perspective adds value by highlighting both universal trends and culture-dependent differences, thereby informing the development of more tailored and effective digital health strategies. Across both populations, Internet use was more common among men than women, consistent with prior literature indicating that men often turn to the Internet for health-related queries, particularly when seeking confidential or anonymous information on sensitive issues (Bidmon & Terlutter, 2015; Chang & Yang, 2021; Ek, 2015; Link & Baumann, 2023). However, other studies show women’s engagement with online health information is generally higher due to their role as primary caregivers, particularly among pregnant women. The present findings align with these patterns, suggesting that gender differences in online health information use are context-dependent and may be shaped by cultural expectations (Bidmon & Terlutter, 2015; Ek, 2015).

In this study, Swiss participants were older and reported more chronic pain, while non communicable diseases such as hypertension and diabetes predominated among Qataris, patterns that mirror broader epidemiology. European data consistently show chronic pain rising with age, with point prevalences commonly in the 12–48% range, aligning with higher pain burden in older Swiss cohorts; Swiss cohort work likewise links pain reporting to age in the general population (Aebischer et al., 2022; Rometsch et al., 2025). In contrast, Qatar’s most recent STEPwise survey shows substantial burden of chronic diseases such as diabetes ∼18% and hypertension ∼24% consistent the finding from this study. The greater use of chronic medications among Swiss participants in our study is expected given their older age profile, as international reviews show polypharmacy rises markedly with age and multimorbidity (Pazan & Wehling, 2021).

Both Swiss and Qatari patients reported high access to digital devices, though Qatari participants were significantly more likely to use them at work, reflecting the country’s high smartphone penetration and strong ICT infrastructure. Qatari patients prioritised health information searches more than Swiss patients, who more often sought general information. Similar trends are seen in the UK (97%), (Moreland et al., 2015) Jeddah, Saudi Arabia (92.7%), (Alghamdi et al., 2019; Chereka et al., 2025)and Malaysia (80%), where health-related searches dominate online activity, compared with 55% across the EU. (Di Novi et al., 2024) Such differences may reflect variations in digital connectivity, health literacy priorities, and cultural attitudes towards seeking medical information online.

Qatari patients were more likely to search for specific diseases, treatments, and healthcare facilities, whereas Swiss patients sought information on health insurance more frequently. The primary motivations for online health searches in both populations were convenience, speed, and availability. However, Swiss patients placed more emphasis on the fact that online information is often free, possibly reflecting cultural or systemic differences in healthcare financing. While most patients sought information for themselves rather than for relatives, Qatari participants were significantly more likely to discuss their findings with doctors, suggesting a higher degree of physician engagement. Swiss patients, in contrast, more often discussed findings with relatives, and to a lesser extent with pharmacists or nurses. These patterns may be influenced by cultural norms regarding the doctor–patient relationship and the perceived authority of healthcare providers (Di Novi et al., 2024; Gallagher & Doherty, 2009; Liu et al., 2024; Liu, 2022).

Compared to Swiss patients who reported little to no impact, Qatari patients more often reported that online health information positively influenced their health decisions, prompted further questions, and improved wellbeing. Qatari patients predominantly searched for topics such as medication risks, alternative therapies, and supplements, while Swiss patients were more interested in over-the-counter drugs. These contrasting patterns are likely shaped by differences in language access, source trust, and pharmacist availability. During hospitalisation, Swiss patients searched their current medications more, whereas Qataris sought complementary therapies, highlighting a preference for integrative or holistic options. These findings are broadly consistent with previous studies published in Qatar, with studies on pregnant women demonstrating heavy reliance on Google and social media for obtaining information on diet and fetal development (Al-Dahshan et al., 2022; Choudhury et al., 2016).

Despite several disparities, both countries consistently prioritised speed and availability, relying mainly on general search engines over specialised medical sites. This underscores the need for healthcare systems in both countries to provide accessible, credible, and culturally tailored online health resources to better guide patient decision-making and reduce the risks of misinformation. Both populations expressed a desire for guidance on improving online searches and evaluating information quality, recognising the challenge of navigating vast and variable online content. Qatari patients showed a stronger preference for video-based content and for official secure websites detailing medication side effects or providing a channel for patient questions. Swiss patients were more interested in curated lists of recommended websites provided by healthcare professionals, either at discharge or during care. These findings are consistent with other studies (McMullan, 2006, 2020).

The findings also highlight the need to improve online health engagement by leveraging hospital Wi-Fi, enhancing digital literacy, and offering culturally tailored strategies such as structured clinician–patient discussions in Qatar and encouraging information sharing in Switzerland. Multilingual, institution-endorsed portals could improve trust and quality, with Arabic video-based resources suiting Qatari needs and evidence-graded written materials meeting Swiss expectations. Training healthcare providers to address patients’ emotional responses to online information is essential to support appropriate interpretation and action.

A key strength of this study is its large sample size of 820 hospitalised patients, which provides sufficient statistical power and enhances the robustness of cross-country comparisons. The inclusion of participants from two countries on different continents Switzerland and Qatar offers unique insights into how cultural, sociodemographic, and healthcare system differences shape online health information–seeking behaviours. This comparative approach enables identification of variations in search priorities, perceived impacts, and preferences, which can guide the development of culturally tailored digital health interventions.

This study has several limitations, notably, the non-response rates were not recorded, which may have introduced bias. The cross-sectional design limits the ability to draw causal inferences between patient characteristics and online behaviours. Self-reported data introduces potential recall and social desirability biases, particularly in sensitive areas such as emotional responses or interest in alternative therapies. As the study was conducted among hospitalised patients, findings may not fully represent community-based populations, as hospitalisation can alter information needs and access patterns. Additionally, differences in internet infrastructure, language accessibility, and healthcare system organisation between Switzerland and Qatar may have influenced the observed trends. The study did not evaluate the accuracy or quality of the information accessed, which limits interpretation of its potential clinical impact. Finally, as data collection occurred in 2016, the findings should be interpreted with caution, as patterns of internet use, digital literacy, and access to health resources may have evolved considerably since then.

## Conclusion

This study demonstrates the growing trend in use of online health information among patients with diverse socioeconomic, sociocultural and linguistic diversities. Patients in both countries predominantly accessed online information for searching health information, confirmation of diagnosis, participating in support groups, interacting with health professionals, second opinion etc. Further studies are required to assess the effect of patient engagement, more specifically around behaviour, utilisation, and potential outcomes and to systematically evaluate the validity and reliability of health information available online.
